# Crimean-Congo Hemorrhagic Fever Virus in Hyalommid Ticks, Northeastern Kenya

**DOI:** 10.3201/eid1708.102064

**Published:** 2011-08

**Authors:** Rosemary Sang, Joel Lutomiah, Hellen Koka, Albina Makio, Edith Chepkorir, Caroline Ochieng, Santos Yalwala, James Mutisya, Lilian Musila, Jason H. Richardson, Barry R. Miller, David Schnabel

**Affiliations:** Author affiliations: Kenya Medical Research Institute, Nairobi, Kenya (R. Sang, J. Lutomiah, E. Chepkorir);; US Army Medical Research Unit Nairobi (H. Koka, A. Makio, C. Ochieng, S. Yalwala, J. Mutisya, L. Musila, J.H. Richardson, D. Schnabel);; Centers for Disease Control and Prevention, Fort Collins, Colorado, USA (B.R. Miller)

**Keywords:** Arbovirus, ticks, Crimean-Congo hemorrhagic fever, species, livestock, viruses, vector-borne infections, zoonoses, dispatch

## Abstract

As part of ongoing arbovirus surveillance, we screened ticks obtained from livestock in northeastern Kenya in 2008 to assess the risk for human exposure to tick-borne viruses. Of 1,144 pools of 8,600 *Hyalomma* spp. ticks screened for Congo-Crimean hemorrhagic fever virus by reverse transcription PCR, 23 pools were infected, demonstrating a potential for human exposure.

Crimean-Congo hemorrhagic fever virus (CCHFV), a member of the genus *Nairovirus*, family *Bunyaviridae*, causes hemorrhagic disease in humans with a >30% case-fatality rate. The virus was first described in 1944 in the Crimea in the former Soviet Union and later was found to be similar to a virus isolated in 1956 in the Belgian Congo (*1*).

Domestic ruminants are infected through tick bites and are able to infect more ticks to perpetuate the virus (*2**,**3*). The virus may be transmitted to humans by the bite of an infected tick or by contact with body fluids from an infected animal or person (*4*). The main vectors of CCHFV are ticks in the genus *Hyalomma*, family *Ixodidae*, with other ixodid ticks and ticks from the family *Argasidae* also contributing to transmission (*2*). The virus is transovarially transmitted among ticks (*2**,**3*); consequently, ticks are also reservoirs of CCHFV.

In Kenya, CCHFV has been detected on only 2 occasions: in *Rhipicephalus pulchellus* ticks collected in the 1970s from a dying sheep in a veterinary laboratory in the town of Kabete outside Nairobi (*2*) and from a person with Crimean-Congo hemorrhagic fever in western Kenya in October 2000 (*5*). Evidence of CCHFV activity in Kenya is limited, and although tick-borne arbovirus surveillance in Kenya has demonstrated circulation of a range of viruses, to our knowledge, detection of CCHFV has not been reported (*6**,**7*).

Crimean-Congo hemorrhagic fever is a substantial public health threat because of the associated high mortality rate (30%–60%), the potential for person-to-person transmission, the unavailability of a licensed vaccine, and the limited treatment options for infected persons (*3**,**4*). Entomologic surveillance is valuable for assessing the risk for human exposure and for identifying so-called hot spots for focused preventive action to minimize the effects of virus outbreaks. As part of ongoing entomologic arbovirus surveillance conducted by the United States Army Medical Research Unit in Kenya and the Kenya Medical Research Institute, ticks were collected from livestock in the semi-arid areas of Kenya, where intense pastoralist farming is practiced, to assess the risk to the community for tick-borne arbovirus exposure.

## The Study

Ticks were sampled in the villages of Diiso and El-Humow and at the livestock market and abattoirs in Garissa District, North Eastern Province of Kenya, during April–May 2008 (Figure). Garissa District is in a semi-arid to arid ecologic zone that receives sporadic rainfall from March to May; vegetation consists primarily of *Acacia-Commiphora* bushes. Its population is largely composed of nomadic herders who travel between districts in northern Kenya in search of water and pasture (*8*).

**Figure:**
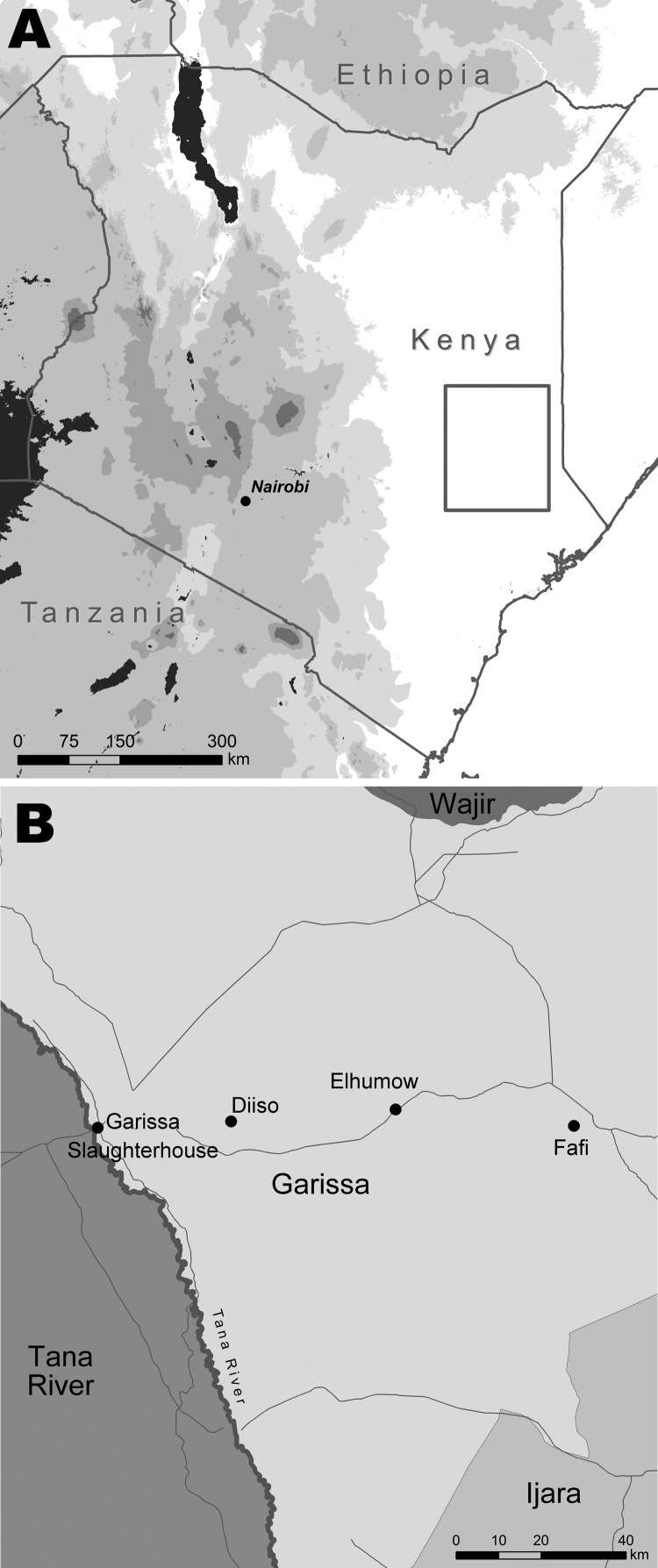
Location of Garissa District (A, box) in North Eastern Province, Kenya, and tick collection sites (B).

Ticks were picked by hand from infested livestock, stored in labeled sterile vials, and transported in liquid nitrogen to the Kenya Medical Research Institute laboratory. In the laboratory, ticks were washed in sterile water, rinsed first with 70% ethanol, and then rinsed with minimum essential medium containing antimicrobial agents (100 U/mL penicillin, 100 μg/mL streptomycin, and 1 μL/mL amphotericin B). They were identified to species by using taxonomic keys (*9**,**10*) and pooled in groups of 2 to 10 by species, sex, collection date and site, and host. The tick pools were homogenized by using 90-mesh alundum sand in a prechilled, sterile mortar and pestle with 1.6 mL–2 mL ice-cold bovine albumin 1 medium (1× medium 199 with Earle salts, 1% bovine albumin, 100 U/mL penicillin, 100 μg/mL streptomycin, and 1 μL/mL amphotericin B) under high containment. The homogenates were clarified by centrifugation at 1,500 rpm for 15 min at 4°C, and supernatants were stored at –80°C.

Viral RNA was extracted from tick homogenates by using Trizol-LS (Invitrogen, Carlsbad, CA, USA) reagent, according to the manufacturer’s instructions. RNA was screened by reverse transcription PCR (*11*) to amplify a 536-bp fragment of the gene encoding for the nucleocapsid protein in the small (S) segment of the CCHFV genome by using the following primers (*12*): CCHF F2 (5′-TGGACACCTTCACAAACTC-3′) and R3 (5′-GACAAATTCCCTGCACCA-3′), positions 135–153 and 653- 670, respectively, on the reference strain CCHFV 10200.

Electrophoresis of the PCR products was performed by using 1% agarose gels in Tris-acetate-EDTA buffer containing ethidium bromide; product bands were visualized and documented with the Canon UVP PhotoDoc-It gel imaging system (UVP, LLC, Upland, CA, USA) mounted with a digital camera. The PCR products of a subset of 4 of the CCHFV-positive homogenates were purified by using the QIAquick PCR Purification Kit (QIAGEN Sciences, Germantown, MD, USA), according to the manufacturer’s instructions, and sequenced by using the BigDye Terminator version 3.1 Cycle Sequencing Kit (Applied Biosystems, Foster City, CA, USA) and the ABI 3730 and automated 3130xl Genetic Analyzer (Applied Biosystems). The sequences were analyzed by using the Basic Local Alignment Search Tool (BLAST; http://blast.ncbi.nlm.nih.gov/Blast.cgi) and the GenBank database to confirm the identity of the virus. Data (including tick species, collection site, animal host, and virologic test results) were entered into an Excel database (Microsoft Corp., Redmond, WA, USA) and analyzed by using pivot tables. A total of 8,600 ticks, of 3 genera and 8 species, were sampled primarily from camels, cattle, goats, and sheep, principally *Hyalomma rufipes* and *Hy. truncatum*. Ticks of the genus *Hyalomma* were sampled 3× more frequently in Diiso than in El-Humow (Table). CCHFV was detected in 23 *Hyalomma* spp. tick pools from Diiso and the Garissa slaughterhouse, including 4 pools of *Hy. rufipes* (3 from cattle and 1 from a camel), 18 pools of *Hy. truncatum* (14 from cattle and 4 from camels), and 1 unidentified *Hyalomma* species (Table) in which single DNA bands corresponding to the predicted 536-bp PCR product were detected.

**Table:** Ticks species sampled from different livestock animals from 4 sites within Garissa District, northeastern Kenya, and CCHFV infection detected from tick pools, April–May 2008*

Site and animal	Tick species	No. ticks	No. pools tested	No. CCHFV-positive pools
El-Humow village				
Goat	*Amblyomma variegatum*	64	16	0
Goat	*Am. gemma*	47	6	0
Cattle	*Am. lepidum*	48	6	0
Camel	*Rhipicephalus appendiculatus*	94	12	0
Camel	*Hyalomma rufipes*	17	3	0
Cattle	*Hy. rufipes*	124	16	0
Sheep	*Hy. rufipes*	50	7	0
Camel	*Hy. truncatum*	22	3	0
Cattle	*Hy. truncatum*	623	81	0
Sheep	*Hy. truncatum*	24	3	0
Cattle	*Hyaloma* sp.	73	10	0
Cattle	*Rh. pulchellus*	748	94	0
Sheep	*Rh. pulchellus*	162	21	0
Diiso village				
Cattle	*Hy. rufipes*	160	20	3
Camel	*Hy. truncatum*	1,034	132	4
Cattle	*Hy. truncatum*	1,513	191	14
Camel	*Hyalomma* sp.	191	24	0
Cattle	*Hyalomma* sp.	192	59	1
Camel	*Rh. pulchellus*	168	21	0
Cattle	*Rh. pulchellus*	1,297	163	0
Goat	*Rh. pulchellus*	420	53	0
Livestock market				
Cattle	*Hy. truncatum*	266	34	0
Cattle	*Hyalomma* sp.	7	1	0
Slaughterhouse				
Camel	*Hy. rufipes*	90	12	1
Camel	*Hy. truncatum*	642	81	0
Camel	*Hyalomma* sp.	76	19	0
Camel	*Rh. pulchellus*	448	56	0
Totals		8,600	1,144	23

*CCHFV, Congo-Crimean hemorrhagic fever virus.

## Conclusions

The detection of CCHFV in pools of *Hyalomma* spp. ticks from Diiso village and the Garissa District slaughterhouse provides strong evidence of CCHFV presence in northeastern Kenya and indicates that CCHFV circulation in Kenya is underestimated. CCHFV was detected only in ticks collected from cattle and a camel. Livestock play a role in the amplification of the virus because the animals become viremic for 7 days (*2**,**3*), during which time they can infect more ticks. Our findings indicate that CCHFV circulates in northeastern Kenya with substantial involvement of camels and cattle. The detection of CCHFV in ticks from camels at the slaughterhouse also suggests the potential of exposure for abattoir workers. The presence of CCHFV among hyalommid ticks in northern Kenya highlights the risk to the resident population and requires the assessment of human exposure. Health care workers must therefore help create awareness among the population and take steps to prepare for and prevent outbreaks.
